# Validation of the General Medication Adherence Scale in Pakistani Patients With Rheumatoid Arthritis

**DOI:** 10.3389/fphar.2020.01039

**Published:** 2020-07-17

**Authors:** Atta Abbas Naqvi, Mohamed Azmi Hassali, Mehwish Rizvi, Ale Zehra, Zeb-un- Nisa, Md. Ashraful Islam, Muhammad Shahid Iqbal, Maryam Farooqui, Mohammad Tarique Imam, Mohammad Akbar Hossain, Irfanullah Khan, Muhammad Zahid Iqbal, Majid Ali, Abdul Haseeb

**Affiliations:** ^1^ Department of Pharmacy Practice, College of Clinical Pharmacy, Imam Abdulrahman Bin Faisal University, Dammam, Saudi Arabia; ^2^ Discipline of Social and Administrative Pharmacy, School of Pharmaceutical Sciences, Universiti Sains Malaysia, Penang, Malaysia; ^3^ Dow College of Pharmacy, Dow University of Health Sciences, Karachi, Pakistan; ^4^ Faculty of Pharmacy, Ziauddin University, Karachi, Pakistan; ^5^ Department of Clinical Pharmacy, College of Pharmacy, Prince Sattam bin Abdulaziz University, Alkharj, Saudi Arabia; ^6^ Department of Pharmacy Practice, Unaizah College of Pharmacy, Qassim University, Qassim, Saudi Arabia; ^7^ Department of Clinical Pharmacy, College of Pharmacy, Umm Al Qura University, Makkah, Saudi Arabia; ^8^ Department of Pharmacology and Toxicology, College of Medicine, Umm Al-Qura University, Makkah, Saudi Arabia; ^9^ Discipline of Clinical Pharmacy, School of Pharmaceutical Sciences, Universiti Sains Malaysia, Penang, Malaysia; ^10^ Department of Clinical Pharmacy and Pharmacy Practice, Faculty of Pharmacy, AIMST University, Bedong, Malaysia

**Keywords:** validation studies, patient compliance, medication persistence, medication adherence, arthritis, rheumatoid, Pakistan

## Abstract

**Objective:**

The aim was to validate the Urdu version of General Medication Adherence Scale (GMAS) in patients with rheumatoid arthritis disease.

**Methods:**

A 2-month (March–April 2019) cross-sectional study was conducted in randomly selected out-patients with rheumatoid arthritis. The sample size was calculated using item-subject ratio of 1:20. The scale was evaluated for factorial, concrete, concurrent, and known group validities. Concrete validity was established by correlating scores of EQ-5D quality of life scale and GMAS adherence score. Concurrent validity was established by correlating the GMAS adherence score with pill count. Analyses for sensitivity were also conducted. Cut-off value was determined through receiver operator curve (ROC), and test–retest method was used to analyze internal consistency and reliability. Data were analyzed through IBM SPSS, IBM AMOS, and MedCalc software. The Urdu version of EQ-5D quality of life questionnaire was used with permission from developers (#ID20884). The study was approved by an ethics committee (#NOV:15).

**Results:**

A total of 351 responses were analyzed. The response rate was 98%. Reliability was in acceptable range, *i.e.*, Cronbach *α* = 0.797. Factorial validity was established by calculation of satisfactory fit indices. Correlation coefficients for concrete and concurrent validities were *ρ* = 0.687, p < 0.01 and *ρ* = 0.779, p < 0.01, respectively. Known group validity was established as significant association of adherence score with insurance and illness duration (p < 0.05) that were reported. Sensitivity of the scale was 94%. Most patients had high adherence (N = 159, 45.3%).

**Conclusion:**

The Urdu version of GMAS demonstrated adequate internal consistency and was validated. These results indicate that it is an appropriate tool to measure medication adherence in Pakistani patients with rheumatoid arthritis.

## Introduction

Rheumatoid arthritis (RA) is a chronic musculoskeletal disease that affects the synovial joints of the body and results in inflammation, stiffness, and arthralgia. It progressively deteriorates the joints and causes joint deformity that reduces mobility. The disease is ranked as one of the leading causes of disability and is linked to productivity losses and economic burden (Guo et al., 2018; Naqvi et al., 2020). Globally, the disease prevalence ranges from 0.5 to 1% with slight variation region-wise. Females are more likely to suffer from the disease as compared to the males. Evidence indicates that polar countries may have a slightly higher prevalence of RA as compared to countries in temperate, torrid, and equatorial regions (Naqvi et al., 2017; Naqvi et al., 2019a; Naqvi et al., 2019d).

The outcomes of RA greatly depend on early initiation of medication therapy and adherence to prescribed medication regimen (Salt et al., 2012). Adherence to medications as advised is considered a cornerstone of disease management and a determinant of positive treatment outcomes as it slows disease progression, reduces pain and inflammation, and preserves joint movement. A systematic review by Li and colleagues reported lower disease activity in adherent patients as compared to non-adherent RA patients (Li et al., 2017). Better management of disease, including medication adherence, could result in lower disease activity, which, in turn, might positively affect QoL. In a ten-year German prospective cohort study involving more than 3,500 patients with rheumatoid arthritis, it was observed that all patients had lower HRQoL compared to the general population (Gerhold et al., 2015). Adherence is essential to maintain a better HRQoL with RA disease in patients. Available evidence indicates that the disease mostly affects the individuals who are working. In worst case, it may contribute to productivity loss, early retirement, economic burden, *etc.* (Naqvi et al., 2017; Naqvi et al., 2020). Hence, monitoring of patients’ adherence is important to select appropriate clinical course and ensure positive treatment outcomes, *i.e.*, lower disease activity and higher HRQoL (Salt et al., 2012; Naqvi et al., 2019d).

Several tools have been formulated to measure medication adherence in chronic diseases. However, few have been validated in patients with RA. The eight and four-item Morisky’s Medication Adherence Scale (MMAS-8 and MMAS-4) and Compliance Questionnaire on Rheumatology (CQR) have been previously validated in patients with RA (Pedersini et al., 2014; Prudente et al., 2016; Xia et al., 2016; Uckun et al., 2017). Several limitations existed as evidence reported that CQR was more sensitive compared to MMAS-4 in documenting adherence. However, CQR was a behavior-oriented scale and had 19 items, making it quite lengthy (Pedersini et al., 2014; Xia et al., 2016). Whereas, MMAS-4 and MMAS-8 were short but expensive as there was a fee for use (Forbes et al., 2018; Naqvi and Hassali, 2019). The shorter five-item version of CQR had better utility however, it addressed adherence related to patient behavior only (Hughes et al., 2013). Adherence is a measurement of medication taking behavior. It not only depends on patients’ behavior, knowledge, and attitudes in general, but is also a result of other determinants such as comorbidity, pill burden, and cost of medicines (Lam and Fresco, 2015). The latter is important in developing countries where patients mostly pay direct treatment costs (Naqvi et al., 2018a).

Pakistan is an economically developing country. The prevalence of RA in Pakistan is between 0.14 and 0.22% with figures for years lived with disability (YLDs) around 29 years and disease adjusted life years to be 40 years. The figures expressed are per 100,000 (Naqvi et al., 2019d; Institute for Health Metrics and Evaluation, 2019). Considering economic repercussions of RA and direct medical expenditure, it was important to utilize a tool that incorporates patient’s medicine taking behaviors, comorbidity, and pill burden as well as cost related non-adherence. Moreover, it is imperative to measure adherence to anti-rheumatic therapy in RA as disease modifying anti-rheumatic drugs (DMARDs) are usually given as a first line of treatment that usually have slow or delayed treatment results. Though, studies have demonstrated that long term adherence to DMARDs would slow disease progression and reduce disease resulted disability and sufferings, there may be a tendency among RA patients to discontinue treatment due to its delayed nature (Xia et al., 2016; Naqvi et al., 2019d).

A novel scale termed as the General Medication Adherence Scale (GMAS) was originally formulated in Urdu language by Naqvi and colleagues that contained 11 items divided into three sections and was validated holistically in patients with chronic illnesses (Naqvi et al., 2018b; Naqvi and Hassali, 2019). However, psychometric properties of GMAS have not been validated in patients with RA.

## Methods

### Aim of the Study

This study aimed to validate psychometric properties of the Urdu version of GMAS in Pakistani patients with RA disease.

### Design

A cross-sectional study with repeated measure was conducted in rheumatology clinics of two tertiary care hospitals of Karachi, Pakistan, for a duration of two months (March–April 2019).

### Participants

Patients with RA were the target population for the study. All adult out-patients who were able to read and write Urdu were considered. Further, patients diagnosed with RA at least 3 months before the study, on long-term therapy with traditional non-biologic disease modifying anti-rheumatic drugs (DMARDs), during implementation phase of adherence were invited. The cut-off time of minimum 3 months of disease duration was considered since patients who were prescribed with medication therapy were usually followed-up after 3 months, *i.e.*, between initiation and discontinuation phases. Therefore, a three month period of implementation phase was considered suitable to assess adherence to medications. According to Vrijens et al. (2012), the implementation phase of adherence is the stage in which the patient continues to take medications from the time of the first dose until the last one. The RA diagnosis was based on ACR/EULAR 2010 criteria (Aydın et al., 2017). Patients with any acute illness, complication or planned surgery were excluded. Patients who did not consent to participate in the study as well as those who returned incomplete questionnaires were left out.

### Enrollment and Randomization

The enrollment of patients was done every morning from 9 am to 11 am. Patients were recruited randomly through a computer-generated list based on their appointment numbers.

### Sample Size Calculation and Research Instrument

The sample size was calculated based on item-respondent theory. A ratio of 1:5 up to 1:20 is considered suitable according to this theory (Osborne and Costello, 2004; Dowrick et al., 2005; Williams and Brown, 2010). Therefore, a ratio of 1:20 was selected and considering 11 items of questionnaire, the required sample size was 220. Considering a 20% drop-out rate, the final sample size was 264.

### Adherence Measure

The research instrument used in the study was the Urdu version of the General Medication Adherence Scale (GMAS) (Naqvi et al., 2018b). The scale consisted of 11 multiple choice questions with four possible options divided into three components. Component 1 measured adherence based on patient behavior while the 2nd component measured adherence based on comorbidity and pill burden. The third measured adherence based on cost. Each item carried an individual score that ranged from 0 to 3. The sum of all 11 individual item scores yield cumulative adherence for a patient that was categorized as high (30–33), good (27–29), partial (17–26), low (11–16), and poor adherence (≤10). The scale, its subscales, and scoring methodology have been previously defined by Naqvi and colleagues (Naqvi et al., 2018b; Naqvi et al., 2019b).

### Statistical Methods for Validation of Research Instrument

#### Factor Analyses

The factor analyses consisted of exploratory factor analysis (EFA), partial confirmatory factor analysis (PCFA), and confirmatory factor analysis (CFA) using structure equation model. Fit indices, namely normed fit index (NFI), comparative fit index (CFI), Tucker Lewis index (TLI), and root mean square error of approximation (RMSEA), were calculated. Factorial validity was established if the values of NFI, CFI, and TLI were 0.9 or higher, and value of RMSEA was <0.08 (Zwick and Velicer, 1986; Pett et al., 2003; Hair et al., 2009). EFA and PCFA were conducted using IBM SPSS version 23, and CFA was carried out using IBM AMOS version 25.

#### Concrete Validity

The concrete validity of GMAS was established by correlating the HRQoL score obtained from Euroqol EQ VAS, with adherence score obtained from GMAS (Cronbach and Meehl, 1955; Strauss and Smith, 2009). Evidence highlights the existence of a relationship between the two in RA disease (Kastien-Hilka et al., 2017). One of the goals of treatment in RA is to improve QoL, and proper adherence to treatment is necessary to achieve treatment goals. Hence, adherence to therapy would lead to an improved QoL (Hromadkova et al., 2015). A positive feedback relationship pertaining to HRQoL and treatment adherence exists as patients with better HRQoL adhere to treatment more often that leads to achievement of better treatment outcomes (Koertge et al., 2003). This motivates patients to follow treatment more actively resulting in improvement in patients’ health and subsequently HRQoL (Bernstein et al., 2002). Thus, HRQoL is considered related to medication adherence in RA.

The Urdu version of EQ-5D quality of life questionnaire was used with permission from the developers (#ID20884) (The EuroQol Group, 1990; Brooks, 1996). Spearman’s rank correlation coefficient (*ρ*) was used to analyze the relationship between EQ VAS score and medication adherence score. EQ VAS (0–100) was a patient-perceived health state at the time of data collection. A score of zero represented worst health state while a score of 100 represented best health state. A value of *ρ* > 0.5 with p-value < 0.05 was considered acceptable (De Vellis, 1991; Salt et al., 2012).

#### Reliability and Internal Consistency

The reliability was analyzed by test–retest method using correlation coefficient (*ρ*) and Cronbach alpha (*α*). Test–retest reliability was evaluated after 3 weeks as recommended by studies (Streiner and Norman, 2003; Wang et al., 2013). A *ρ* value of 0.7 or higher with p-value < 0.05 were considered significant test–retest correlation (De Vellis, 1991; Cohen, 1998). Additionally, an *α* value of 0.5 or greater was considered acceptable (De Vellis, 1991; Cohen, 1998). Composite reliability was measured using McDonald’s coefficient (*ω*). A value of 0.7 or greater for *ω* was considered satisfactory (McDonald, 1999; Trizano-Hermosilla and Alvarado, 2016). Furthermore, item-to-correlation (ITC), range of correlations among items, average correlation value, and intraclass correlation coefficient (ICC) were calculated. A value of 0.5 or greater was considered acceptable for ICC (Lahey et al., 1983; Bowling, 2009; Sushil and Verma, 2010).

#### Known Group Validity

The study had assumptions that medication adherence would be different in known patient groups. Known group validity measures the capability of a psychometric research instrument to distinguish among samples with known characteristics (McConnell et al., 2001). Such patient groups could be defined based on insurance claims and illness duration. It was hypothesized that patients who had insurance would exhibit better adherence compared to patients with no insurance. This hypothesis was based on the evidence that direct medical expenditure may act as a barrier to treatment (Naqvi et al., 2018a). Moreover, it was also assumed that patients who were recently diagnosed with RA would be more adherent compared to those who had diagnosis for longer duration (Brown and Bussell, 2011; Maningat et al., 2013; Marengo and Suarez-Almazor, 2015; Bansilal et al., 2016). The known group validity was evaluated through cross tabulation and significant chi-square (χ^2^) test result (De Vellis, 1991; Cohen, 1998; Kurlander et al., 2009; Iuga and McGuire, 2014). Patients were classified as adherent, *i.e.*, having adherence score ≥ 27 and non-adherent, *i.e.*, with adherence score ≤ 26. Patients with either full or partial insurance were classified as insured while those with no insurance were designated as not insured. For duration of illness, the patients were classified into three groups, *i.e.*, <1 year, between 1 and 3 years, and >3 years.

#### Concurrent Validity

The concurrent validity was analyzed by correlating overall adherence score established by the GMAS with actual patient compliance to therapy after three weeks. Patient compliance to medication therapy was calculated by pill-count based on their medicines taking pattern in last 3 weeks and comparing it with prescribed medications. This was evaluated at follow-up after 3 weeks. Patients were asked to bring their remaining medicines at follow-up visit. Compliance was expressed as a percentage (%) and was calculated based on a formula.

$$C\%=\frac{{ND}_{pt}}{{ND}_{Rx}}\times 100$$

Where *C*_%_ is the (%) compliance, *ND_pt_* is the number of doses assumed to be taken by patient, and *ND_Rx_* denotes the number of doses prescribed to patient. Spearman’s rank correlation coefficient (*ρ*) was used to evaluate concurrent validity. A *ρ* value of 0.7 or greater with p-value < 0.05 was deemed acceptable (Naqvi et al., 2019b).

### Tests to Determine Adequacy of GMAS

A test was conducted to evaluate the ability of GMAS to screen patients based on EQ value index quadrant (76–100) from EQ-5D quality of life scale. The EQ value was calculated as per the scoring criteria provided by EuroQol (The EuroQol Group, 1990). The index value represents a current state of health of a patient which is important in the estimation of quality adjusted life years that range from 0 to 1. A figure of zero represents death while the figure one represents healthy state (Herdman et al., 2011; van Hout et al., 2012). The quadrant 0.76–1.0 denoted HRQoL of patients between satisfactory to best health state that could be achieved with the disease.

The analysis included evaluation of sensitivity, specificity, likelihood ratios, predictive values, and accuracy using the MedCalc Version 19.3.1 statistical software. All values except those for ratios were reported in percentage (%) while 95% confidence interval ranges were reported for all values. Clopper–Pearson method was used to calculate confidence interval ranges for sensitivity, specificity, and accuracy (Altman et al., 2000). Standard logit was used to report confidence interval range for predictive values, while log method was used to calculate likelihood ratio ranges (Zhou et al., 2002; Mercaldo et al., 2007).

### Determination of Cut-Off Values

The scoring methodology defined by Naqvi and colleagues did not contain the cut-off values for designating a patient as adherent or non-adherent (Naqvi et al., 2018b). Though the authors had previously defined cut-off value of GMAS at score of 27 that discriminated between adherent and non-adherent patients, they did not mention the process in their work (Naqvi et al., 2018b). This task was carried out for the first time using receiver operating curve (ROC) and area under the curve (AUC) (Altman and Bland, 1994; Fawcett, 2006). Based on the ROC–AUC analysis a cut-off value with highest value for sensitivity and lowest inverse of specificity was selected.

### Data Analyses

The data were analyzed through IBM SPSS version 23 and IBM AMOS version 25 (Armonk, USA) and MedCalc version 19.3.1 statistical software. Categorical variables were expressed as sample counts (N) and percentages (%), while continuous variables were expressed as mean (X) and standard deviation (SD) and, where applicable, in 95% confidence interval range. Statistical significance was considered at p-value < 0.05.

### Ethics Approval and Consent

The study was approved by the ethics committee of Allied Med Ethics (#NOV:15), Karachi, Pakistan. All patients were asked to provide a written consent to participate before data collection. Patients who provided their consent were included in the study. Those who did not consent to participate where excluded from the study.

## Results

### Patient Information

A total of 357 patients responded to the study, and 351 questionnaires were returned completed and analyzed. The mean age of respondents was 46.6 ± 14.6 years. Most patients were females (N = 260, 74.1%) and married (N = 301, 85.8%). More than half of the patients were educated (N = 252, 71.8%) and had a monthly family income of more than PKR 50,000, *i.e*., >USD 314.72, (N = 190, 54.1%) which is considered a high income. Most patients were involved in household activities (N = 168, 47.9%) and lived in urban localities (N = 243, 69.2%). Slightly less than half of the patients were diagnosed with RA less than one year before the study (N = 166, 47.3%). The average number of medicines on a prescription was between 4 and 5, *i.e.*, 4.88 ± 1.5.

Most commonly used DMARD was hydroxychloroquine (HCQ) (N = 142, 40.5%). Some patients were prescribed with a combination of two DMARDs, *i.e.*, MTX and HCQ (N = 14, 4%), HCQ and leflunomide (N = 25, 7.1%), MTX and leflunomide (N = 11, 3.1%), MTX and sulfasalazine (SSZ) (N = 11, 3.1%), HCQ and SSZ (N = 16, 4.6%), and leflunomide and SSZ (N = 6, 1.7%). Few patients were prescribed with a triple DMARDs therapy, *i.e.*, MTX–HCQ–SSZ (N = 11, 3.1%) and HCQ–leflunomide–SSZ (N = 15, 4.3%). Most patients had no insurance (N = 272, 77.5%) and a third had at least one comorbidity (N = 121, 34.4%). Of those 121 patients who had comorbidity, 50 had diabetes mellitus, 34 had hypertension, 12 patients had asthma, eight had kidney disorders, three had gastrointestinal diseases, two patients had thyroid disease, one had benign prostate hyperplasia and 11 had multimorbidity (**Table 1**).

**Table 1:** Patient demographic information (N = 351).

Patient information	Sample (N)	Percentage (%)
**Gender**		
Male	91	25.9
Female	260	74.1
**Marital status**		
Single	50	14.2
Married	301	85.8
**Education**		
Had at least primary education	252	71.8
No formal education	99	28.2
**Occupation**		
Household	168	47.9
Employed	104	29.6
Self-employed	18	5.1
Retired	15	4.3
Unemployed	46	13.1
Monthly family income*		
Less than PKR 10,000 (<USD 62.94)	3	0.9
Between PKR 10,000 and PKR 25,000 (USD 62.94 and 157.36)	19	5.4
Between PKR 25,000 and PKR 50,000 (USD 157.36 and 314.72)	139	39.6
More than PKR 50,000 (>USD 314.72)	190	54.1
**Residence**		
Urban	243	69.2
Rural	108	30.8
**Duration of illness**		
Less than 1 year	166	47.3
Between 1 and 3 years	120	34.2
Between 3 and 5 years	45	12.8
Between 5 and 10 years	15	4.3
More than 10 years	5	1.4
**DMARDs used for RA**		
Hydroxychloroquine	142	40.5
Methotrexate	53	15.1
Sulfasalazine	29	8.3
Leflunomide	18	5.1
Combination of two DMARDs	83	23.6
Combination of three DMARDs	26	7.4
**Total number of medicines prescribed**		
Between 2 and 4	144	41
Between 5 and 6	157	44.7
Between 7 and 9	50	14.3
**Had at least 1 comorbidity**		
Yes	121	34.4
No	230	58.2
**Insurance**		
Full insurance	28	8
Partial insurance	51	14.5
No insurance	272	77.5

*Average monthly income in Pakistan was PKR 35,662 (USD 224.47) (Naqvi et al., 2018a), 1 USD equals 158.87 at the time of this writing.

### Adherence to Medication Therapy

Mean adherence score was 28.6 ± 3.2 out of 33 (median 29). Most patients (N = 159, 45.3%) had high adherence while a third (N = 112, 31.9%) had good adherence. Less than a quarter (N = 79, 22.5%) had partial adherence, and one patient (0.3%) had low adherence. The response distribution and details of adherence with respect to DMARDs’ use are tabulated in **Tables 2** and **3**, respectively.

**Table 2:** Response distribution.

GMAS items	Item description	Participants’ response (N/%)			
		1	2	3	4
1	Difficulty in remembering to take medications	1 (0.3)	16 (4.6)	97 (27.6)	237 (67.5)
2	Forgetting medications due to busy schedule, travel, and other events	–	–	94 (26.8)	257 (73.2)
3	Discontinuing medication when feeling well	5 (1.4)	4 (1.1)	86 (24.5)	256 (72.9)
4	Stop taking medications due to adverse effects	5 (1.4)	2 (0.6)	116 (33)	228 (65)
5	Stop taking medications without informing the doctor	4 (1.1)	6 (1.7)	85 (24.2)	256 (72.9)
6	Discontinuing medicines due to other medicines for additional disease	3 (0.9)	90 (25.6)	–	258 (73.5)
7	Find it hassle to remember medications due to medication regime complexity	4 (1.1)	–	129 (36.8)	218 (62.1)
8	Missing medicines due to progression of disease and addition of new medicines in the last month	–	2 (0.6)	160 (45.6)	189 (53.8)
9	Altering medication regimen, dose, and frequency	6 (1.7)	11 (3.1)	81 (23.1)	253 (72.1)
10	Discontinuing medications because they are not worth the money	66 (18.8)	23 (6.6)	135 (38.5)	127 (36.2)
11	Finding it difficult to buy medicines because they are expensive	1 (0.3)	7 (2)	56 (16)	287 (81.8)

1 = Always, 2 = Mostly, 3 = Sometimes, and 4 = Never.

**Table 3:** Patients’ adherence to specific DMARD therapy.

Patients on DMARD therapy	Adherence interpretation (N/%)*			
	High	Good	Partial	Low
Hydroxychloroquine (N = 142)	70 (49.3)	35 (24.6)	36 (25.3)	1 (0.8)
Methotrexate (N = 53)	18 (34)	26 (49)	9 (17)	0 (0)
Sulfasalazine (N = 29)	9 (31)	11 (38)	9 (31)	0 (0)
Leflunomide (N = 18)	10 (55.6)	6 (33.3)	2 (11.1)	0 (0.8)
Combination therapy with two DMARDs (N = 83)	40 (48.2)	26 (31.3)	17 (20.5)	0 (0)
Combination therapy with three DMARDs (N = 26)	12 (46.2)	8 (30.8)	6 (23)	0 (0)

*Adherence interpretation: High = 30 – 33, Good = 27 – 29, Partial = 17 – 26, Low = 11 – 16.

### Factor Analyses

**Figure 1:**
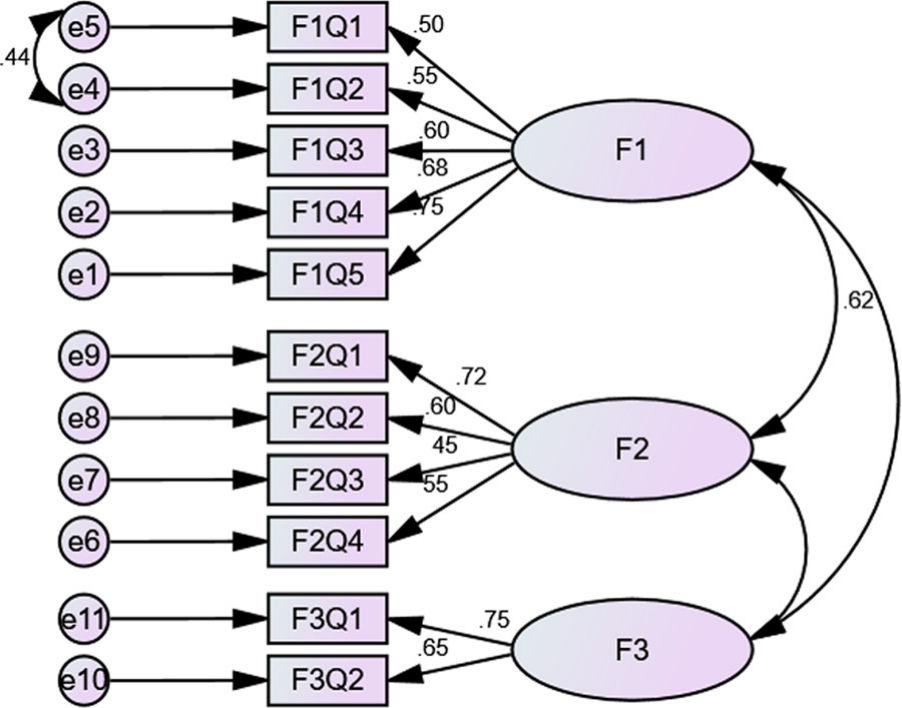
CFA with fit indices.

The factor analyses consisted of EFA and PCFA. EFA was done using principal component analysis (PCA) with oblimin rotation. The value for the Kaiser–Meyer–Olkin measure of sampling adequacy was reported at 0.772, *i.e.*, >0.7, and Bartlett’s test of sphericity was significant, *i.e.*, χ2 value of 814.772, d = 55, and p < 0.0001. A three-factor solution was obtained with eigenvalues > 1 and explained an average variance of 55%. For the purpose of statistical analyses, items that had a loading value above 0.3 on one component and less than 0.3, *i.e.*, non-salient loading, on another component were considered a single factor. Factor one (F1), *i.e.*, Patient behaviors, consisted of five items, factor two (F2), *i.e.*, Comorbidity and pill burden, had four, while factor three (F3), *i.e.*, Out-of-pocket expenditure, had two items loaded (**Figure 1**).

PCFA was conducted using Maximum Likelihood Analysis (MLA) with the same rotation with factors fixed at 3. The implied model χ^2^ value was reported at 67.039, df = 25. A normal distribution curve was reported for non-salient factor loadings with mean score of 0.3. Fit indices, *i.e.*, NFI = 0.92, TLI = 0.9, and CFI = 0.94 were greater than 0.9, while RMSEA was less than 0.07 that highlights good factor structure fit.

Additionally, structure equation modeling was conducted, and CFA revealed that the model was fit (χ^2^ = 126.289, df = 4, p < 0.000). The χ^2^/df = 3.08 and the fit indices were: GFI = 0.93, TLI = 0.9, AGFI = 0.9, and CFI = 0.9, *i.e.*, ≥0.9 while RMSEA was 0.77, *i.e.*, <0.08. (**Figure 1**).

### Concrete Validity

**Figure 2:**
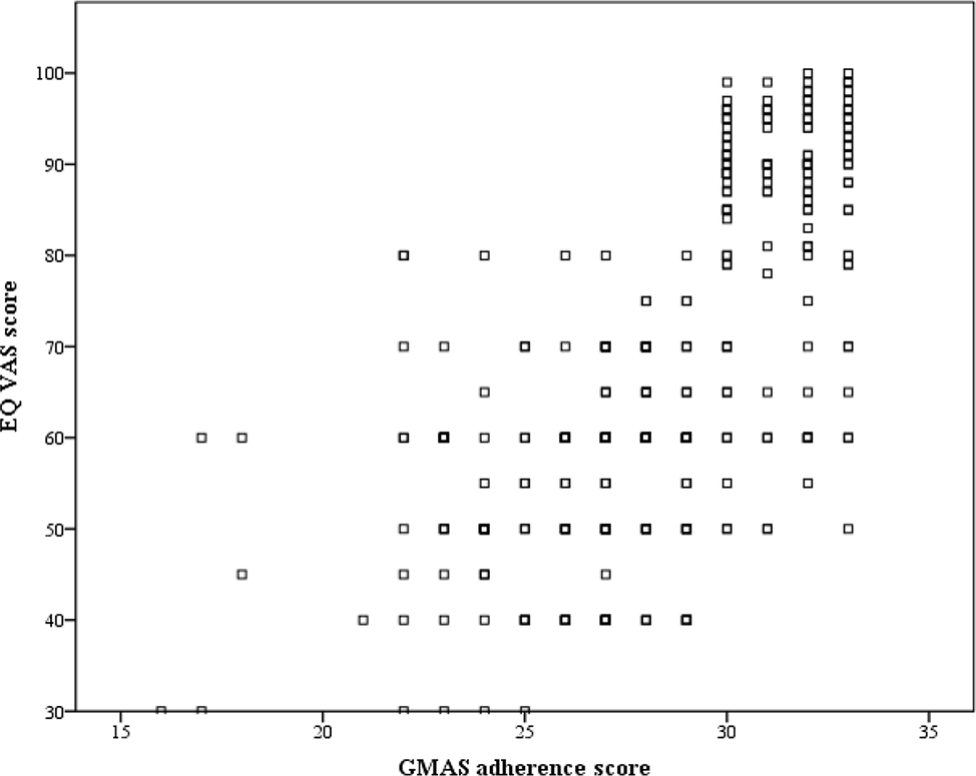
Correlation between GMAS adherence score and EQ VAS.

There was a positive correlation (*ρ* = 0.687, p-value < 0.0001), and hence concrete validity of GMAS was established (**Figure 2**).

### Reliability and Internal Consistency

The GMAS had a value of 0.797 for Cronbach (*α*) and 0.87 for McDonald’s coefficient (*ω*). All items were positively correlated except item 10 that appeared to negatively correlate with item 7. The corrected item-to-total correlation ranged from 0.21 to 0.6. No significant item redundancy was present. The average correlation value was 0.27. Intraclass correlation coefficient was 0.797 and ranged from 0.763 to 0.827 for 95% CI. The test–retest reliability was reported at 0.875 with p-value less than 0.0001.

### Known Group Validity

The χ^2^ value was 4.57 and p-value was <0.05, *i.e.*, 0.03, for cross-tabulation of adherence with insurance, while χ^2^ value was 12.52 and p-value was <0.01, *i.e.*, 0.002, for cross-tabulation of adherence with illness duration. Hence, known group validity was established (**Table 4**).

**Table 4:** Cross-tabulation between GMAS adherence score and demographic variables.

Variables		GMAS adherence	
		Adherent	Non-adherent
**Insurance**			
**Insured**	Count (Expected Count)	68 (61)	11 (18)
	% within Insurance (% within GMAS adherence score)	86.1 (25.1)	13.9 (13.8)
**Not insured**	Count (Expected Count)	203 (210)	69 (62)
	% within Insurance (GMAS adherence score)	74.6 (74.9)	25.4 (86.3)
**Duration of illness**			
**<1 year**	Count (Expected Count)	141 (128.2)	25 (37.8)
	% within Illness duration (GMAS adherence score)	84.9 (52)	15.1 (31.3)
**1 to 3 years**	Count (Expected Count)	88 (92.6)	32 (27.4)
	% within Illness duration (GMAS adherence score)	73.3 (32.5)	26.7 (40)
**>3 years**	Count (Expected Count)	42 (50.2)	23 (14.8)
	% within Illness duration (GMAS adherence score)	64.6 (15.5)	35.4 (28.7)

Adherent = GMAS score ≥ 27, Non-adherence = GMAS score ≤26.

### Concurrent Validity

**Figure 3:**
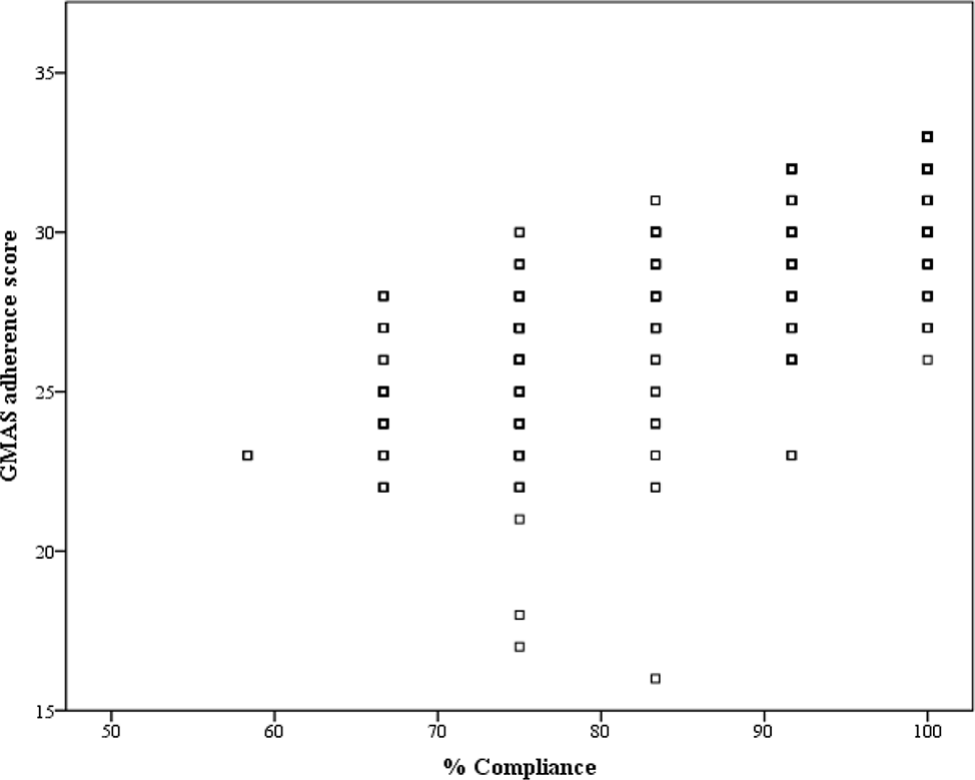
Correlation between GMAS adherence score and percentage compliance.

The correlation coefficient (*ρ*) was 0.779 with p-value<0.001, and hence, concurrent validity was established (**Figure 3**).

### Tests to Determine Adequacy of GMAS

The GMAS was 94.5% [CI 95%: 86.56–98.49%] sensitive and 83.3%, [CI 95%: 58.58–96.42%] specific in screening patients with high to good adherence based on EQ index value (0.76–1.0) quadrant. The positive and negative likelihood ratios were 5.67 [CI 95%: 2.02–15.96] and 0.07 [CI 95%: 0.02–0.17] respectively. The (+) predictive value was 95.8%, [CI 95%: 89.1–98.5%] while the (−) predictive value was 78.9%, [CI 95%: 58.59–90.86%]. Accuracy was reported at 92.3%, [CI 95%: 84.79–96.85%].

### Determination of Cut-Off Values

**Figure 4:**
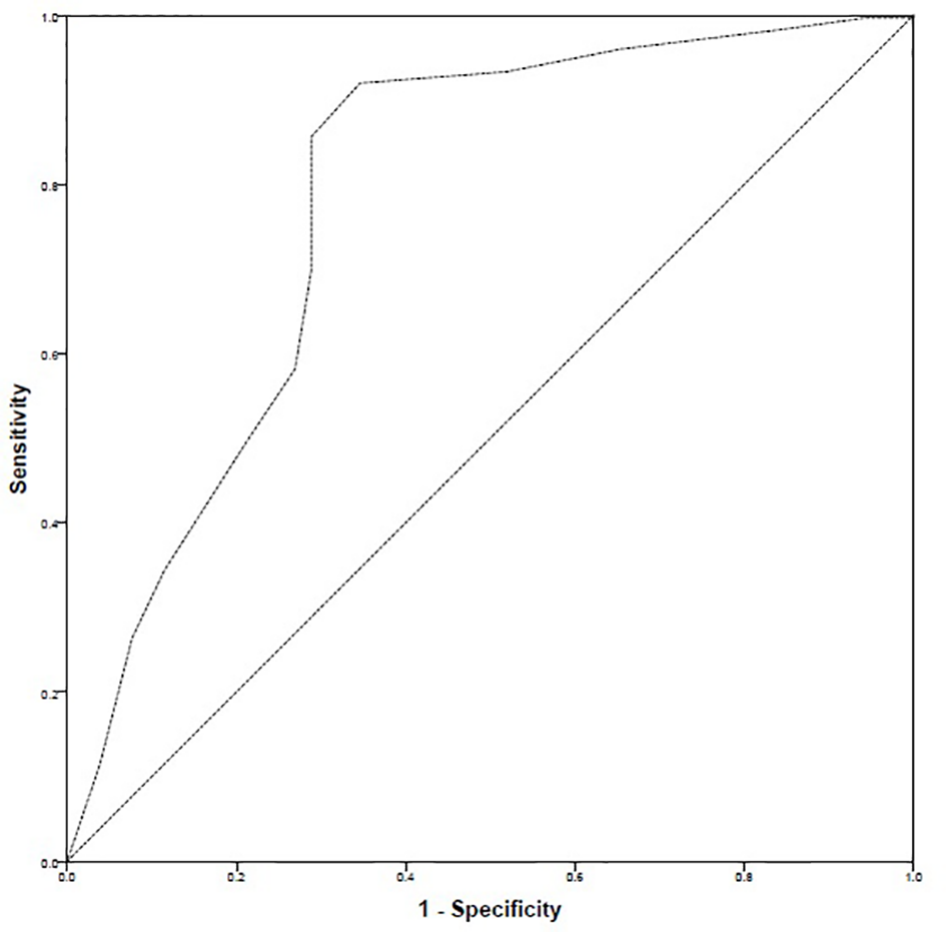
ROC curve.

The ROC curve highlighted that there were 299 positive cases and 52 negative cases. The positive cases meant that patients had high–good adherence. The ROC–AUC was 78.3% *i.e.*, 0.783 ± 0.041 [95% CI: 0.703–0.863]. Based on ROC–AUC coordinates, a cut-off value of 27 was identified to discriminate patients with high–good adherence from the rest of the sample. The sensitivity at cut-off value of 27 was 0.856 while the inverse of specificity was 0.288 (**Figure 4**).

## Discussion

The validation process of GMAS included factor analyses, assessment of reliability and internal consistency as well as establishment of validities. Factor analyses revealed a 3-factor structure (**Figure 1**) with satisfactory fit indices. This finding was in line with previous validation studies of GMAS in Pakistani and Saudi patients (Naqvi et al., 2018b; Naqvi et al., 2019a; Naqvi et al., 2019c). According to Osborne and Costello, a factor with less than three items is generally weak and unstable, especially when the sample size is relatively small (Osborne and Costello, 2005). But in practice, factor should be retained if it can interpret the domain in a meaningful way (Worthington and Whittaker, 2006; Kenny, 2016). A factor with two items is only considered when the items are highly correlated and at the same time fairly uncorrelated with others, by imposing equality constraints on the factor loadings (Yong and Pearce, 2013). In the current study, factor 3 was considered as it fulfilled the statistical criteria. The sample size was large enough, and the observed correlation between items was relatively higher, and at the same time, a weak correlation among other items. Most importantly, factor 3 was retained due to its importance in the questionnaire.

Two reliability measures *i.e.*, Cronbach (*α*) and McDonald’s (*ω*), were used. The values obtained for *α* and *ω* were satisfactory and indicated good internal consistency. Furthermore, reliability was assessed by the test–retest method (Lahey et al., 1983). The reliability values obtained for GMAS were higher than those reported for MMAS-8 and CQR (de Klerk et al., 1999; Zhou et al., 2002; Salt et al., 2012). It is important to achieve a satisfactory reliability as Salt and colleagues observed a low *α* value for the Medication Adherence Scale (MAS) and concluded that MAS would be inappropriate to use in RA patients (Salt et al., 2012). The concrete validity of GMAS was assessed by correlating the adherence score of patients with the EQ VAS score. EQ VAS is a part of the EQ-5D quality of life tool that is a reliable tool to measure health related quality of life (HRQoL). Since a patient’s mobility and productivity are most affected by the disease, HRQoL would be significantly impacted (Aydın et al., 2017). Moreover, clinical evidence mentions that adherence to medications slows down disease progression (Aydın et al., 2017; Li et al., 2017; Guo et al., 2018). Therefore, correlation of adherence score with EQ VAS scores was deemed suitable. We found a significantly moderate-to-strong correlation between the two variables. This occurrence was in line with available clinical evidence (Oyoo et al., 2011). Hence, the concrete validity of GMAS was established.

The known group validity was established for GMAS. It was observed that most patients with insurance had better adherence while most patients with no insurance appeared non-adherent. The result was significant, and this finding highlights that direct treatment cost may act as a barrier to treatment. This was in line with previous studies conducted in this population (Hussain et al., 2014; Gillani et al., 2018). Similarly, patients with recently diagnosed RA were more adherent as compared to those diagnosed earlier. This finding was consistent with previous reports that with the passage of time patients may become less compliant to their prescribed therapy (Brown and Bussell, 2011; Maningat et al., 2013; Bansilal et al., 2016).

A systematic review by Forbes and colleagues identified that no self-reporting tool could be considered as a standard to measure medication adherence. They recommended a combination of objective and subjective measures for documenting adherence (Forbes et al., 2018). Therefore, we used an objective method, *i.e.*, pill count along with subjective measure, *i.e.*, self-reporting GMAS tool. The adherence level from the pill count was expressed as percentage compliance and was correlated with GMAS adherence score to evaluate its concurrent validity (Altman et al., 2000). The correlation was significantly strong, and hence, concurrent validity was established.

The scale demonstrated high sensitivity and specificity in screening patients with high–good adherence, based on EQ value index score between 0.76 and 1.0. The index value represents a current state of health of a patient that is important in the estimation of quality adjusted life years (QALYs) that range from 0 to 1. A figure of zero represented death while the figure of one represented a healthy state (Herdman et al., 2011; van Hout et al., 2012). The quadrant 0.76–1.0 denoted HRQoL of patients between satisfactory and best health state that could be achieved with the disease. The results revealed that sensitivity was above 94% while specificity was more than 83%. This meant that GMAS was able to correctly identify majority of the patients with better health state who were adherent. A high positive likelihood ratio was obtained that highlighted that patients with high–good adherence were almost six times more likely to have a better HRQoL. The negative likelihood ratio of 0.07 was observed which entailed that there was a decreased likelihood for patients with low adherence to have better HRQoL. A high (+) predictive value obtained indicated that most patients who were identified in the better HRQoL quadrant as adherent were true positives. At the same time, a high (−) predictive value indicated that very few patients who were identified as non-adherent were in the better HRQoL quadrant (Altman et al., 2000). This study highlighted that less than a quarter of the patients (22.5%) were partially adherent to medications. This was consistent with a study conducted in Pakistan to document medication adherence in RA patients which reported similar figure of 23% of RA patient who were non-adherent (Arshad et al., 2016).

Naqvi and colleagues had previously defined cut-off value of GMAS at a score of 27 that discriminated between adherent and non-adherent patients but did not mention the process in their work (Naqvi et al., 2018b). Therefore, we conducted the ROC analysis using AUC to identify a cut-off and observed that the cut-off value of 27 previously defined by Naqvi and colleagues had a sensitivity >85% and an inverse of specificity less than 29% (Naqvi et al., 2018b). Hence, this was in line with the previous findings of Naqvi et al. (2018), and a cut-off score of 27 was selected (Naqvi et al., 2018b).

The study is novel as no medication adherence tool has been previously validated in this population. MMAS-8 was the only validated scale available for documenting adherence to medications in Pakistani patients with hypertension (Saleem et al., 2012). However, it was not validated in RA patients. Besides, it does not estimate cost related non-adherence which is an important factor in measuring adherence in this population (Naqvi et al., 2018b; Naqvi and Hassali, 2019). Another strength of this tool is that it was available in Urdu language that made it easier for patients to understand and provide their response.

Following the methodology of Naqvi and colleagues, the GMAS was sampled in RA patients using item-response theory to calculate the sample size (De Vellis, 1991; Cohen, 1998). The sampling was random unlike previous MMAS-8 validation study that used convenience sample and gathered responses from a nominal sample of patients (Saleem et al., 2012). The response rate achieved was high that indicates that patient did not find it difficult to respond to GMAS. These aspects could be considered as strengths of this study. Response rate is an important aspect as questionnaires with low response rate might warrant reconsideration in their text. This aspect was highlighted in a study on CQR validation in which participants had confusion regarding some items of the tool that resulted in low factor loadings, and hence, modification was recommended before handing the tool in patients (Salt et al., 2012). The tool was significantly shortened later in a study (Hughes et al., 2013).

The GMAS measures adherence to medications in the implementation phase of adherence in which patients are already taking their medicines. According to De Geest and colleagues, common forms of non-adherence to therapy during implementation phase are missing or skipping a dose, reducing quantity of dose, decreasing frequency of dose, and/or taking a drug holiday (De Geest et al., 2018). The GMAS had items specifically designed to measure these forms of non-adherence and could only highlight the extent to which patients were adherent/non-adherent to a prescribed therapy. The scale does not measure adherence in the initiation phase where patients take the first dose of prescribed medicines as there is no item that addresses on-time, delayed, or incomplete initiation. Moreover, the scale does not have any item that could highlight if the patients discontinue their medication therapy earlier than scheduled.

The availability of GMAS would allow the rheumatologists to document adherence to medications among patients with rheumatoid arthritis. The disease modifying anti-rheumatic drugs (DMARDs) are usually given as a first line of treatment for the disease (Naqvi et al., 2019d). The treatment from DMARDs is usually slow; however, studies have demonstrated that long term adherence to DMARDs would slow disease progression and reduce disease resulted disability and sufferings (Xia et al., 2016). Therefore, adhering to medication would ensure better treatment outcomes. The use of GMAS to evaluate adherence of RA patients would help rheumatologists monitor patients’ treatment progress as well as identify any factors that may act as barriers to DMARD therapy adherence.

This may offer an opportunity to pharmacists to provide behavioral counseling to patients aimed at modifying their medicine taking behavior and improving their selfreported adherence.

## Conclusion

The GMAS had strong internal consistency and established factorial, concrete, concurrent and known group validities. The tool had high sensitivity and specificity. It demonstrated better psychometric properties compared to previously validated tools in RA patients. Besides, the values obtained during analysis were higher than those reported from Urdu version of MMAS-8, the only scale validated in Pakistani population. These properties deem the Urdu version of GMAS an appropriate tool to measure medication adherence in patients with RA. Further studies validating GMAS in other disease population are recommended.

## Data Availability Statement

The raw data supporting the conclusions of this article are property of the healthcare facility. It will be made available by the authors on suitable request subject to approval from healthcare body. The scale and its scoring code could be obtained from corresponding author on request.

## Ethics Statement

The studies involving human participants were reviewed and approved by the ethics committee of Allied Med Ethics (#NOV:15). The patients/participants provided their written informed consent to participate in this study..

## Author Contributions

AN and MAH conceived the idea with AZ, MR, MF, and Z-U-N. The study was designed by AN, AZ, MR, ZN, MAI, and MSI. The data were collected by AN, AZ, MR, Z-U-N, IK, and AH. The data were analyzed by AN, MAI, MSI, MTI, MAK, IK, MZI, MA, and AH. The *Abstract* and *Introduction* were written by AN, AZ, and MR. The *Methods*, *Results*, *Discussion*, and *Conclusion* sections were written, reviewed, and edited by all authors. All authors were involved in the revision of manuscript. The whole work was supervised by MAH. All authors contributed to the article and approved the submitted version.

## Conflict of Interest

The authors declare that the research was conducted in the absence of any commercial or financial relationships that could be construed as a potential conflict of interest.
